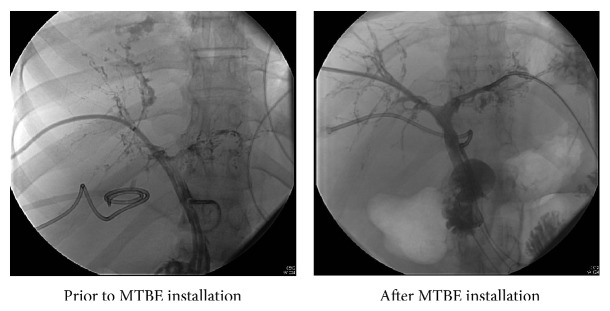# Use of Methyl Tert-Butyl Ether for the Treatment of Refractory Intrahepatic Biliary Strictures and Bile Casts: A Modern Perspective

**DOI:** 10.1155/2015/408175

**Published:** 2015-07-07

**Authors:** Gregory Kim, Saninuj N. Malayaman, Michael Stuart Green

**Affiliations:** Department of Anesthesiology and Perioperative Medicine, Drexel University College of Medicine/Hahnemann University Hospital, 245 N. 15th Street, Suite 7502, MS 310, Philadelphia, PA 19102, USA

## Abstract

Cholelithiasis is a prevalent problem in the United States with 14% or more adults affected. Definitive treatment of cholelithiasis is cholecystectomy. When cholecystectomy yields minimal resolution treatment options include expectant management of asymptomatic gallstones or endoscopic retrograde cholangiopancreatogram. We present a case of intrahepatic biliary casts where surgical option was not possible, interventional radiology was unsuccessful, and methyl tert-butyl ether was used to dissolve the biliary obstruction. Dissolution therapy of gallstones was first reported in 1722 when Vollisnieri used turpentine in vitro. While diethyl ether has excellent solubilizing capacity, its low boiling point limited its use surgically as it vaporizes immediately. Diethyl ether can expand 120-fold during warming to body temperature after injection into the biliary system making it impractical for routine use. The use of dissolution is out of favor due to the success of laparoscopic cholecystectomy. Epidemiological studies have shown the general population should have minimal concerns from passive exposure. Dissolution using MTBE remains a viable option if surgical or endoscopic options are not available. However, because of risks involved to both the patient and the staff, careful multidisciplinary team approach must be undertaken to minimize the risks and provide the best possible care to the patient.

## 1. Introduction

Cholelithiasis, the formation of gallstones, is a prevalent problem in the United States with 14% or more adults affected at some time in their life. Aggregate costs, mostly from surgical and operative procedures, are estimated at $2.2 billion per year [1, 2]. Definitive treatment of cholelithiasis is cholecystectomy, via open or, more recently favored, laparoscopic approach. Because of the gradual progression of gallstone disease, there is an increased prevalence of cholelithiasis and choledocholithiasis in the older population. By age 65, approximately 30% of women have gallstones and by the age 80, 60% of both men and women are affected [3]. While the elderly patient population has increased inherent risks due to presence of comorbid medical conditions, routine use of minimally invasive laparoscopic cholecystectomy has made surgical treatment more acceptable, even in asymptomatic cases, compared to open cholecystectomy [4].

In a patient where surgical cholecystectomy does not resolve disease or when medical comorbid conditions produce a patient too unstable to undergo surgery, possible treatment options include expectant management of an asymptomatic gallstone or endoscopic retrograde cholangiopancreatogram (ERCP). We present a case of intrahepatic biliary casts where surgical option was not possible, interventional radiological options were unsuccessful, and methyl tert-butyl ether (MTBE), a relatively outdated and underutilized compound, was used to dissolve the biliary obstruction.

## 2. Case Presentation

A 56-year-old female with a past medical history of COPD, GERD, and bipolar disease was transferred to our institution following inpatient treatment at two outside hospitals. She had been admitted to the first hospital with abdominal pain and found to have gallstone pancreatitis, cholangitis, and* Streptococcus salivarius* bacteremia. While a cholecystectomy was considered, she instead underwent ERCP to remove the stone and a percutaneous cholecystostomy tube was placed by interventional radiology. After three weeks, she was discharged with a course of oral antibiotics.

Two weeks later she was admitted to a different facility with right upper quadrant abdominal pain, nausea, and vomiting. Liver enzymes and total bilirubin were elevated. The patient revealed that she had not taken the full course of oral antibiotics. ERCP was performed with small stones seen. The patient underwent a planned cholecystectomy which began as laparoscopic and was converted to open. This procedure was unsuccessful and a cholecystostomy drain was placed with intraoperative cholangiogram showing an obstruction in the common bile duct. Gastroenterology made the decision to abort the procedure and instead place drains in the biliary tree.

One week later the patient was transferred to our institution for higher acuity of care. An open cholecystectomy with common bile duct exploration and common bile duct repair was performed. Postoperatively labs remained elevated with white blood cell (WBC) 19.1 K/UL, alkaline phosphatase 1060 U/L, total bilirubin 15.79 mg/dL, and direct bilirubin 9.86 mg/dL. A series of cholangiograms revealed intrahepatic biliary strictures and bile casts in the intrahepatic biliary tree. Interventional radiology attempted drainage of bile but was unsuccessful. The patient's WBC count and LFTs continued to rise and she progressed to liver failure. Cardiology consult was obtained but the patient refused a recommended pharmacologic stress test. A psychology consult commented that the patient was not psychologically stable for a liver transplant.

Given the past procedural and surgical attempts to drain biliary casts, the decision was made to attempt to dissolve the casts using methyl tert-butyl ether (MTBE). The patient was brought into the operating room where she was given general anesthesia via an endotracheal tube. MTBE was directly injected into the biliary system with 30 mL ether injected in the right biliary system and 46 mL injected into the left biliary system. Following MTBE treatment, there was marked improvement of the right biliary duct system and modest improvement of the left biliary duct system (Figure 1). The patient's alkaline phosphatase decreased to 590 U/L, total bilirubin 14.44 mg/dL, and direct bilirubin 8.79 mg/dL, but WBC remained at 15.3 K/UL. She continued to have persistent Gram-negative rod infection for which she remained on long term antibiotics. Twenty days following MTBE injection, and a little over 2 months after being admitted to our institution, the patient was discharged to a rehabilitation center.

## 3. Discussion

Dissolution therapy of gallstones was first reported in 1722 when Vollisnieri used turpentine in vitro. Other solvents used for dissolution of choledocholithiasis tried with some success were diethyl ether, a potent cholesterol solvent and chloroform. While diethyl ether has excellent solubilizing capacity, its low boiling point of 34.5°C limited its use surgically as it vaporizes almost immediately. As a solvent, diethyl ether can expand 120-fold as it is warmed to body temperature after injection into the biliary system making it impractical for routine use [5].

In 1978 monooctanoin (MO) was used to treat biliary stones and since has been used via delivery through a transhepatically placed catheter, T-tube, or endoscopically placed retrograde catheter [6, 7]. Dissolution time for MO was often prolonged on the order of days.

In order to find a faster acting solvent, methyl tertiary butyl ether (MTBE) was tried with success in 1986 [5, 6]. The use of dissolution has fallen out of favor for most cases of cholelithisis due to the success and acceptable risk benefit profile of laparoscopic cholecystectomy as perceived by both physicians and patients.

Methyl tertiary butyl ether (MTBE), mostly used as an additive to improve gasoline octane, has higher boiling point of 55.2°C which makes it a favorable candidate for a solvent [5, 8, 9]. MTBE has a similar dissolving capacity as diethyl ether as well as similar pharmacokinetics and toxicity [6]. Because of government regulation on fuel composition and concern for toxicity from high atmospheric concentrations, MTBE has been studied extensively. MTBE is rapidly distributed into the bloodstream following inhalation or ingestion and almost exclusively exhaled with minimal undergoing metabolism in the liver [9, 10]. Epidemiological studies have shown that the general population should have minimal concerns from passive exposure of MTBE from automobile emissions. Those whose work entails exposure to higher concentration of MTBE showed little to no adverse health effects [9].

Since the use of MTBE for dissolution of gallstones in the 1980s, multiple case studies have documented successful dissolution of gallstones [10–14]. Leuschner et al. showed a dissolution rate of 96.6% in 120 patients with a low rate of complication and toxic side effects [14]. A larger survey in Europe of 803 patients from 21 institutions showed excellent dissolution rates with fewer side effects [11]. Common complaints included nausea, upper abdominal pain, duodenitis, and hemolysis [6]. Without proper ventilation, the odor did cause light headedness and nausea from the staff in one study [6] yet the odor from the European survey showed no issues from patient or staff [11].

### 3.1. Case-Specific Concerns

Because of the inexperience of our providers with using MTBE, a multidisciplinary panel which included gastroenterology, interventional radiology, surgery, and anesthesiology was formed to discuss the possible risks associated with the patient as well as the safety of those taking care of her. Although there have been documented cases following the initial publication of successful MTBE use, dissolution of stones has waned in favor of endoscopic or interventional radiologic techniques. Strict guidelines and protocols were not established so judicious use of MTBE was necessary. The margin of error was relatively small in our patient due to her state of critical illness with ongoing bacteremia and liver failure. Systemic toxicity was also a concern because, while the gallbladder can act as a chamber to facilitate dissolving of the stones and limit the amount of systemic absorption, our patient had casts in the bile ducts and had a previous cholecystectomy.

Multiple steps were taken to ensure the safety of the patient and the staff. The procedure was performed in a general OR suite which allowed for the best ventilation to remove the MTBE-generated fumes. Safety personnel were present throughout the entire case. Ambient room temperature was reduced and humidity was increased. All unnecessary potential sources of sparks including phones and pagers were removed from the operating room. The patient was intubated and a low fraction of inspiratory oxygen (FiO_2_) was administered to decrease the potential for any airway fires. Once the biliary duct system was identified, MTBE was directly administered in slow and incremental doses to avoid systemic toxicity. On the right duct system, a total of 30 mL of MTBE was administered over the course of 45 minutes in 5 mL increments. On the left duct system, a total of 46 mL of MTBE was administered over the course of 67 minutes in 10 mL increments. Following the procedure, the patient remained intubated and was taken to the intensive care unit where she was further closely monitored for any complications.

## 4. Conclusion

In conclusion, dissolution using MTBE, while not commonly performed, still remains a viable option if surgical or endoscopic options are not available. However, because of risks involved to both the patient and the staff, careful multidisciplinary team approach must be undertaken to minimize the risks and provide the best possible care to the patient.

## Figures and Tables

**Figure 1:**